# Diethyl 4-[4-(dimethyl­amino)phen­yl]-2,6-dimethyl-1,4-dihydro­pyridine-3,5-dicarboxyl­ate

**DOI:** 10.1107/S1600536810003508

**Published:** 2010-02-06

**Authors:** Xiao-Ling Ji, Wang-Bin Sun, Qian Zhang, Yan-Ping Cao, Ming-Sheng Bai

**Affiliations:** a College of Life Science, Yulin University, Yulin, Shaanxi 719000, People’s Republic of China; b College of Chemistry and Chemical Engineering, Yulin University, Yulin, Shaanxi 719000, People’s Republic of China; c College of Life Science, Ningxia University, Yinchuan 750021, People’s Republic of China

## Abstract

In the title compound, C_21_H_28_N_2_O_4_, the dihydro­pyridine ring adopts a flattened boat conformation. The mean plane of the dihydro­pyridine ring and the attached benzene ring form a dihedral angle of 85.1 (1) Å. One of two ethyl fragments is disordered between two conformations in a 0.67 (4):0.33 (4) ratio. In the crystal structure, mol­ecules related by translation along the *a* axis are linked into chains *via* inter­molecular N—H⋯O hydrogen bonds.

## Related literature

For the pharmacological activity of compounds containing substituted 1,4-dihydro­pyridine ring systems, see: Triggle *et al.*(1980 ▶); Henry (2004 ▶). For a related structure, see Sun *et al.* (2006 ▶).
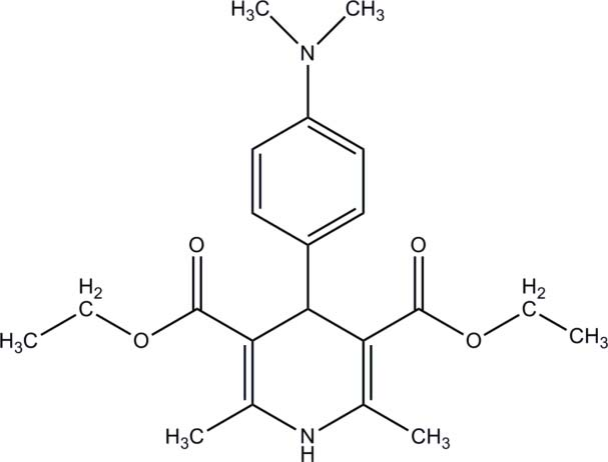


## Experimental

### 

#### Crystal data

C_21_H_28_N_2_O_4_
*M* *_r_* = 372.45
Monoclinic, 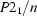

*a* = 7.5023 (7) Å
*b* = 14.9797 (14) Å
*c* = 18.0691 (19) Å
β = 91.021 (1)°
*V* = 2030.3 (3) Å^3^
*Z* = 4
Mo *K*α radiation
μ = 0.08 mm^−1^
*T* = 298 K
0.49 × 0.48 × 0.47 mm

#### Data collection

Bruker SMART APEX CCD area-detector diffractometer
Absorption correction: multi-scan (*SADABS*; Sheldrick, 1996 ▶) *T*_min_ = 0.960, *T*_max_ = 0.961
10007 measured reflections
3572 independent reflections
1680 reflections with *I* > 2σ(*I*)
*R*_int_ = 0.067

#### Refinement

*R*[*F* ^2^ > 2σ(*F* ^2^)] = 0.061
*wR*(*F* ^2^) = 0.202
*S* = 1.02
3572 reflections
270 parameters
H-atom parameters constrained
Δρ_max_ = 0.19 e Å^−3^
Δρ_min_ = −0.18 e Å^−3^

### 

Data collection: *SMART* (Siemens, 1996 ▶); cell refinement: *SAINT* (Siemens, 1996 ▶); data reduction: *SAINT*; program(s) used to solve structure: *SHELXS97* (Sheldrick, 2008 ▶); program(s) used to refine structure: *SHELXL97* (Sheldrick, 2008 ▶); molecular graphics: *SHELXTL* (Sheldrick, 2008 ▶); software used to prepare material for publication: *SHELXTL*.

## Supplementary Material

Crystal structure: contains datablocks I, global. DOI: 10.1107/S1600536810003508/cv2687sup1.cif

Structure factors: contains datablocks I. DOI: 10.1107/S1600536810003508/cv2687Isup2.hkl

Additional supplementary materials:  crystallographic information; 3D view; checkCIF report

## Figures and Tables

**Table 1:** Hydrogen-bond geometry (Å, °)

*D*—H⋯*A*	*D*—H	H⋯*A*	*D*⋯*A*	*D*—H⋯*A*
N1—H1⋯O4^i^	0.86	2.18	3.036 (4)	173

Symmetry code: (i) 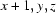.

**Fig. 1.**
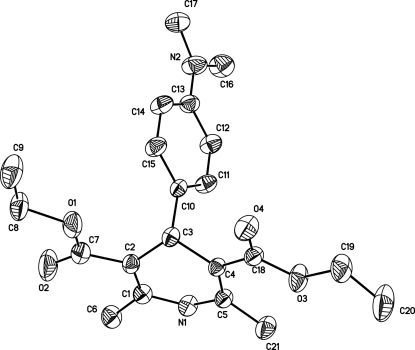
A view of (I) showing the atomic numbering scheme and 30% probability displacement ellipsoids. Only major parts of disordered atoms are shown. H atoms omitted for clarity.

## supplementary crystallographic information

### Comment

Substituted 1,4-dihydropyridine ring system exhibit diverse pharmacological activities (Triggle *et al.*, 1980). In addition, several of these compounds were discovered to be highly selective ligands for adenosine receptors, which were recently recognized as potential targets for the development of new drugs for the treatment of Parkinson's disease, hypoxia/ischemia, asthma, kidney disease, epilepsy, and cancer (Henry *et al.*, 2004). In continuation of our ongoing program directed to the development of pyridine chemistry, we present here the crystal structure of the title compound, (I).

In  (I) (Fig. 1), the bond lengths an angles are normal and are comparable to the values observed in similar compound ethyl 5-carboxy-2,6-dimethyl-4-(4-nitrophenyl) -1,4-dihydropyridine-3-carboxylate (Sun *et al.*, 2006). The carboxylate and methyl groups lie to the  each side  of  the dihydropyridine ring. The dihedral angle between the planes C2/C3/C4 and C1/N1/C5 is  36.9 (3) Å. The dihydropyridine ring adopts a flattened boat conformation and the plane of the base of the boat (C1/C2/C4/C5) forms a dihedral angle of 83.73 (13) Å with the benzene ring.

In the crystal structure, intermolecular N—H···O hydrogen bonds (Table 1) link the molecules into chains propagated in direction [100].

### Experimental

4-(N, N-dimethyl)benzaldehyde (10.0 mmol), 20 ml methanol, ethyl acetoacetate (20 mmol) and NH_4_HCO_3_ (6 mmol)  were mixed in 50 ml flash. After refluxing 3 h, the resulting mixture was cooled to room temperature, and recrystalized from methanol, and afforded the title compound as a crystalline solid. Elemental analysis: calculated for C_21_H_28_N_2_O_4_: C 67.72, H 7.58, N 7.52%; found: C 67.82, H 7.73, N 7.43%.

### Refinement

All H atoms were placed in geometrically idealized positions ( C-H of methyl distances is 0.96 Å , C-H of methylene distances is 0.97 Å , C-H of methine distances is 0.98 Å  and aromatic C-H distances is 0.93 Å, N-H distances is 0.86 Å) and treated as riding on their parent atoms, with U_iso_(H) = 1.2 U-1.5_eq_(C, N) . Atoms C_8_, C_9_  were treated as disordered between two positions, with refined occupancies of 0.33 (4) and 0.67 (4).

### Crystal data

**Table:**

C_21_H_28_N_2_O_4_	*F*(000) = 800
*M**_r_* = 372.45	*D*_x_ = 1.218 Mg m^−^^3^
Monoclinic, *P*2_1_/*n*	Mo *K*α radiation, λ = 0.71073 Å
*a* = 7.5023 (7) Å	Cell parameters from 1665 reflections
*b* = 14.9797 (14) Å	θ = 2.6–20.9°
*c* = 18.0691 (19) Å	µ = 0.08 mm^−^^1^
β = 91.021 (1)°	*T* = 298 K
*V* = 2030.3 (3) Å^3^	Needle, red
*Z* = 4	0.49 × 0.48 × 0.47 mm

### Data collection

**Table:**

Bruker SMART APEX CCD area-detector diffractometer	3572 independent reflections
Radiation source: fine-focus sealed tube	1680 reflections with *I* > 2σ(*I*)
graphite	*R*_int_ = 0.067
phi and ω scans	θ_max_ = 25.0°, θ_min_ = 1.8°
Absorption correction: multi-scan (*SADABS*; Sheldrick, 1996)	*h* = −8→8
*T*_min_ = 0.960, *T*_max_ = 0.961	*k* = −11→17
10007 measured reflections	*l* = −21→21

### Refinement

**Table:**

Refinement on *F*^2^	Primary atom site location: structure-invariant direct methods
Least-squares matrix: full	Secondary atom site location: difference Fourier map
*R*[*F*^2^ > 2σ(*F*^2^)] = 0.061	Hydrogen site location: inferred from neighbouring sites
*wR*(*F*^2^) = 0.202	H-atom parameters constrained
*S* = 1.01	*w* = 1/[σ^2^(*F*_o_^2^) + (0.0653*P*)^2^ + 1.4769*P*] where *P* = (*F*_o_^2^ + 2*F*_c_^2^)/3
3572 reflections	(Δ/σ)_max_ = 0.001
270 parameters	Δρ_max_ = 0.19 e Å^−^^3^
0 restraints	Δρ_min_ = −0.18 e Å^−^^3^

### Special details

**Table:**

Geometry. All esds (except the esd in the dihedral angle between two l.s. planes) are estimated using the full covariance matrix. The cell esds are taken into account individually in the estimation of esds in distances, angles and torsion angles; correlations between esds in cell parameters are only used when they are defined by crystal symmetry. An approximate (isotropic) treatment of cell esds is used for estimating esds involving l.s. planes.
Refinement. Refinement of F^2^ against ALL reflections. The weighted R-factor wR and goodness of fit S are based on F^2^, conventional R-factors R are based on F, with F set to zero for negative F^2^. The threshold expression of F^2^ > 2sigma(F^2^) is used only for calculating R-factors(gt) etc. and is not relevant to the choice of reflections for refinement. R-factors based on F^2^ are statistically about twice as large as those based on F, and R- factors based on ALL data will be even larger.

### Fractional atomic coordinates and isotropic or equivalent isotropic displacement parameters (Å^2^)

**Table:**

	*x*	*y*	*z*	*U*_iso_*/*U*_eq_	Occ. (<1)
N1	1.0562 (4)	0.1513 (2)	1.06111 (16)	0.0535 (9)	
H1	1.1638	0.1618	1.0758	0.064*	
N2	0.6296 (5)	0.5330 (2)	0.8972 (2)	0.0785 (11)	
O1	0.6518 (4)	0.0907 (2)	0.86907 (16)	0.0810 (10)	
O2	0.9341 (5)	0.0681 (3)	0.84207 (18)	0.1139 (14)	
O3	0.6311 (3)	0.1834 (2)	1.20424 (14)	0.0693 (9)	
O4	0.4439 (3)	0.1727 (2)	1.10883 (14)	0.0679 (9)	
C1	1.0290 (5)	0.1244 (2)	0.9891 (2)	0.0495 (10)	
C2	0.8620 (5)	0.1274 (2)	0.96009 (18)	0.0464 (9)	
C3	0.7131 (5)	0.1728 (2)	1.00311 (17)	0.0436 (9)	
H3	0.6020	0.1402	0.9931	0.052*	
C4	0.7524 (4)	0.1684 (2)	1.08551 (17)	0.0414 (9)	
C5	0.9222 (5)	0.1625 (3)	1.11124 (18)	0.0474 (9)	
C6	1.1942 (5)	0.0920 (3)	0.9527 (2)	0.0677 (12)	
H6A	1.1736	0.0334	0.9327	0.102*	
H6B	1.2902	0.0896	0.9885	0.102*	
H6C	1.2250	0.1321	0.9135	0.102*	
C7	0.8257 (7)	0.0925 (3)	0.8863 (2)	0.0648 (12)	
C8	0.624 (3)	0.0697 (13)	0.7887 (8)	0.077 (4)	0.67 (4)
H8A	0.6810	0.1141	0.7582	0.092*	0.67 (4)
H8B	0.6723	0.0115	0.7771	0.092*	0.67 (4)
C9	0.428 (2)	0.0708 (15)	0.7756 (8)	0.095 (5)	0.67 (4)
H9A	0.3732	0.0268	0.8065	0.142*	0.67 (4)
H9B	0.4022	0.0574	0.7246	0.142*	0.67 (4)
H9C	0.3820	0.1288	0.7873	0.142*	0.67 (4)
C8'	0.557 (5)	0.036 (2)	0.814 (2)	0.079 (9)	0.33 (4)
H8'1	0.4525	0.0074	0.8345	0.094*	0.33 (4)
H8'2	0.6338	−0.0093	0.7929	0.094*	0.33 (4)
C9'	0.508 (6)	0.107 (3)	0.7585 (16)	0.098 (10)	0.33 (4)
H9'1	0.4027	0.1381	0.7745	0.146*	0.33 (4)
H9'2	0.4844	0.0805	0.7111	0.146*	0.33 (4)
H9'3	0.6041	0.1491	0.7547	0.146*	0.33 (4)
C10	0.6878 (4)	0.2688 (2)	0.97650 (17)	0.0420 (9)	
C11	0.7898 (5)	0.3382 (3)	1.0039 (2)	0.0577 (11)	
H11	0.8738	0.3263	1.0411	0.069*	
C12	0.7721 (5)	0.4242 (3)	0.9787 (2)	0.0619 (11)	
H12	0.8445	0.4686	0.9990	0.074*	
C13	0.6486 (5)	0.4466 (3)	0.9232 (2)	0.0553 (10)	
C14	0.5446 (5)	0.3772 (3)	0.8956 (2)	0.0629 (11)	
H14	0.4592	0.3888	0.8589	0.076*	
C15	0.5659 (5)	0.2911 (3)	0.9218 (2)	0.0584 (11)	
H15	0.4947	0.2462	0.9015	0.070*	
C16	0.7323 (7)	0.6039 (3)	0.9282 (3)	0.0911 (16)	
H16A	0.7142	0.6064	0.9806	0.137*	
H16B	0.6959	0.6593	0.9060	0.137*	
H16C	0.8563	0.5937	0.9189	0.137*	
C17	0.4833 (7)	0.5557 (3)	0.8486 (3)	0.0834 (15)	
H17A	0.4969	0.5257	0.8021	0.125*	
H17B	0.4815	0.6190	0.8407	0.125*	
H17C	0.3735	0.5374	0.8705	0.125*	
C18	0.5961 (5)	0.1746 (2)	1.1321 (2)	0.0467 (9)	
C19	0.4839 (5)	0.1922 (3)	1.2541 (2)	0.0735 (14)	
H19A	0.3974	0.1452	1.2448	0.088*	
H19B	0.4253	0.2493	1.2466	0.088*	
C20	0.5528 (7)	0.1859 (4)	1.3293 (3)	0.113 (2)	
H20A	0.6072	0.1285	1.3367	0.170*	
H20B	0.4570	0.1932	1.3632	0.170*	
H20C	0.6400	0.2319	1.3377	0.170*	
C21	0.9918 (5)	0.1636 (3)	1.1893 (2)	0.0708 (13)	
H21A	0.9342	0.2103	1.2162	0.106*	
H21B	1.1181	0.1739	1.1895	0.106*	
H21C	0.9677	0.1071	1.2123	0.106*	

### Atomic displacement parameters (Å^2^)

**Table:**

	*U*^11^	*U*^22^	*U*^33^	*U*^12^	*U*^13^	*U*^23^
N1	0.0339 (17)	0.075 (2)	0.0513 (19)	−0.0034 (15)	0.0033 (15)	−0.0005 (16)
N2	0.089 (3)	0.055 (2)	0.091 (3)	−0.002 (2)	−0.019 (2)	0.017 (2)
O1	0.085 (2)	0.086 (2)	0.071 (2)	0.0062 (18)	−0.0216 (17)	−0.0345 (17)
O2	0.108 (3)	0.169 (4)	0.065 (2)	0.033 (3)	0.006 (2)	−0.044 (2)
O3	0.0432 (16)	0.118 (3)	0.0468 (17)	0.0008 (15)	0.0103 (13)	−0.0104 (15)
O4	0.0372 (16)	0.109 (2)	0.0575 (17)	0.0035 (15)	0.0019 (13)	0.0050 (15)
C1	0.043 (2)	0.056 (2)	0.050 (2)	−0.0050 (19)	0.0102 (18)	0.0038 (19)
C2	0.051 (2)	0.048 (2)	0.041 (2)	−0.0027 (18)	0.0047 (18)	−0.0003 (17)
C3	0.039 (2)	0.053 (2)	0.0386 (19)	−0.0056 (17)	0.0000 (16)	0.0004 (17)
C4	0.037 (2)	0.050 (2)	0.0371 (19)	0.0003 (17)	0.0017 (16)	0.0013 (16)
C5	0.040 (2)	0.060 (3)	0.043 (2)	0.0017 (18)	0.0032 (18)	0.0021 (18)
C6	0.056 (3)	0.084 (3)	0.064 (3)	0.004 (2)	0.020 (2)	−0.003 (2)
C7	0.070 (3)	0.070 (3)	0.054 (3)	0.007 (2)	−0.007 (2)	−0.009 (2)
C8	0.081 (10)	0.092 (9)	0.058 (7)	−0.008 (6)	−0.009 (6)	−0.029 (6)
C9	0.079 (10)	0.134 (13)	0.071 (7)	−0.020 (7)	−0.011 (6)	−0.032 (7)
C8'	0.085 (18)	0.079 (17)	0.071 (16)	−0.012 (11)	−0.005 (13)	−0.023 (13)
C9'	0.10 (3)	0.12 (2)	0.075 (14)	−0.004 (16)	−0.022 (15)	0.003 (14)
C10	0.041 (2)	0.051 (2)	0.0346 (19)	−0.0020 (18)	0.0004 (16)	0.0033 (16)
C11	0.054 (2)	0.059 (3)	0.059 (2)	−0.006 (2)	−0.0139 (19)	0.009 (2)
C12	0.063 (3)	0.055 (3)	0.066 (3)	−0.012 (2)	−0.016 (2)	0.006 (2)
C13	0.058 (3)	0.054 (3)	0.053 (2)	0.003 (2)	0.002 (2)	0.010 (2)
C14	0.061 (3)	0.066 (3)	0.061 (3)	0.000 (2)	−0.018 (2)	0.010 (2)
C15	0.062 (3)	0.056 (3)	0.056 (2)	−0.005 (2)	−0.018 (2)	0.002 (2)
C16	0.106 (4)	0.065 (3)	0.102 (4)	−0.010 (3)	−0.005 (3)	0.013 (3)
C17	0.100 (4)	0.069 (3)	0.081 (3)	0.014 (3)	−0.007 (3)	0.019 (3)
C18	0.041 (2)	0.057 (3)	0.041 (2)	0.0039 (18)	0.0010 (18)	0.0046 (18)
C19	0.048 (3)	0.113 (4)	0.059 (3)	0.006 (2)	0.018 (2)	−0.006 (2)
C20	0.095 (4)	0.186 (7)	0.059 (3)	0.004 (4)	0.022 (3)	−0.018 (3)
C21	0.048 (2)	0.112 (4)	0.052 (2)	0.013 (2)	−0.005 (2)	−0.001 (2)

### Geometric parameters (Å, °)

**Table:**

N1—C1	1.374 (4)	C8'—C9'	1.51 (7)
N1—C5	1.375 (4)	C8'—H8'1	0.9700
N1—H1	0.8600	C8'—H8'2	0.9700
N2—C13	1.384 (5)	C9'—H9'1	0.9600
N2—C16	1.421 (5)	C9'—H9'2	0.9600
N2—C17	1.434 (5)	C9'—H9'3	0.9600
O1—C7	1.336 (5)	C10—C15	1.376 (5)
O1—C8'	1.47 (2)	C10—C11	1.377 (5)
O1—C8	1.497 (11)	C11—C12	1.372 (5)
O2—C7	1.208 (5)	C11—H11	0.9300
O3—C18	1.332 (4)	C12—C13	1.393 (5)
O3—C19	1.444 (4)	C12—H12	0.9300
O4—C18	1.210 (4)	C13—C14	1.388 (5)
C1—C2	1.350 (5)	C14—C15	1.381 (5)
C1—C6	1.495 (5)	C14—H14	0.9300
C2—C7	1.453 (5)	C15—H15	0.9300
C2—C3	1.531 (5)	C16—H16A	0.9600
C3—C4	1.514 (4)	C16—H16B	0.9600
C3—C10	1.528 (5)	C16—H16C	0.9600
C3—H3	0.9800	C17—H17A	0.9600
C4—C5	1.352 (5)	C17—H17B	0.9600
C4—C18	1.459 (5)	C17—H17C	0.9600
C5—C21	1.495 (5)	C19—C20	1.447 (6)
C6—H6A	0.9600	C19—H19A	0.9700
C6—H6B	0.9600	C19—H19B	0.9700
C6—H6C	0.9600	C20—H20A	0.9600
C8—C9	1.48 (3)	C20—H20B	0.9600
C8—H8A	0.9700	C20—H20C	0.9600
C8—H8B	0.9700	C21—H21A	0.9600
C9—H9A	0.9600	C21—H21B	0.9600
C9—H9B	0.9600	C21—H21C	0.9600
C9—H9C	0.9600		
			
C1—N1—C5	124.2 (3)	H9'1—C9'—H9'3	109.5
C1—N1—H1	117.9	H9'2—C9'—H9'3	109.5
C5—N1—H1	117.9	C15—C10—C11	115.7 (3)
C13—N2—C16	120.9 (4)	C15—C10—C3	122.1 (3)
C13—N2—C17	120.2 (4)	C11—C10—C3	122.2 (3)
C16—N2—C17	117.9 (4)	C12—C11—C10	122.6 (4)
C7—O1—C8'	129.0 (13)	C12—C11—H11	118.7
C7—O1—C8	110.6 (8)	C10—C11—H11	118.7
C8'—O1—C8	33.4 (12)	C11—C12—C13	121.7 (4)
C18—O3—C19	118.7 (3)	C11—C12—H12	119.2
C2—C1—N1	118.6 (3)	C13—C12—H12	119.2
C2—C1—C6	127.7 (4)	N2—C13—C14	121.7 (4)
N1—C1—C6	113.7 (3)	N2—C13—C12	122.2 (4)
C1—C2—C7	120.1 (4)	C14—C13—C12	116.1 (4)
C1—C2—C3	119.9 (3)	C15—C14—C13	121.0 (4)
C7—C2—C3	120.0 (3)	C15—C14—H14	119.5
C4—C3—C10	111.8 (3)	C13—C14—H14	119.5
C4—C3—C2	110.4 (3)	C10—C15—C14	122.9 (4)
C10—C3—C2	110.2 (3)	C10—C15—H15	118.5
C4—C3—H3	108.1	C14—C15—H15	118.5
C10—C3—H3	108.1	N2—C16—H16A	109.5
C2—C3—H3	108.1	N2—C16—H16B	109.5
C5—C4—C18	124.6 (3)	H16A—C16—H16B	109.5
C5—C4—C3	120.5 (3)	N2—C16—H16C	109.5
C18—C4—C3	114.9 (3)	H16A—C16—H16C	109.5
C4—C5—N1	118.5 (3)	H16B—C16—H16C	109.5
C4—C5—C21	129.4 (3)	N2—C17—H17A	109.5
N1—C5—C21	112.1 (3)	N2—C17—H17B	109.5
C1—C6—H6A	109.5	H17A—C17—H17B	109.5
C1—C6—H6B	109.5	N2—C17—H17C	109.5
H6A—C6—H6B	109.5	H17A—C17—H17C	109.5
C1—C6—H6C	109.5	H17B—C17—H17C	109.5
H6A—C6—H6C	109.5	O4—C18—O3	120.7 (3)
H6B—C6—H6C	109.5	O4—C18—C4	124.1 (3)
O2—C7—O1	120.4 (4)	O3—C18—C4	115.1 (3)
O2—C7—C2	126.8 (4)	O3—C19—C20	108.4 (4)
O1—C7—C2	112.8 (4)	O3—C19—H19A	110.0
C9—C8—O1	106.0 (14)	C20—C19—H19A	110.0
C9—C8—H8A	110.5	O3—C19—H19B	110.0
O1—C8—H8A	110.5	C20—C19—H19B	110.0
C9—C8—H8B	110.5	H19A—C19—H19B	108.4
O1—C8—H8B	110.5	C19—C20—H20A	109.5
H8A—C8—H8B	108.7	C19—C20—H20B	109.5
O1—C8'—C9'	99 (3)	H20A—C20—H20B	109.5
O1—C8'—H8'1	111.9	C19—C20—H20C	109.5
C9'—C8'—H8'1	111.9	H20A—C20—H20C	109.5
O1—C8'—H8'2	111.9	H20B—C20—H20C	109.5
C9'—C8'—H8'2	111.9	C5—C21—H21A	109.5
H8'1—C8'—H8'2	109.6	C5—C21—H21B	109.5
C8'—C9'—H9'1	109.5	H21A—C21—H21B	109.5
C8'—C9'—H9'2	109.5	C5—C21—H21C	109.5
H9'1—C9'—H9'2	109.5	H21A—C21—H21C	109.5
C8'—C9'—H9'3	109.5	H21B—C21—H21C	109.5

### Hydrogen-bond geometry (Å, °)

**Table:**

*D*—H···*A*	*D*—H	H···*A*	*D*···*A*	*D*—H···*A*
N1—H1···O4^i^	0.86	2.18	3.036 (4)	173

Symmetry codes: (i) *x*+1, *y*, *z*.
